# Cyclic response of ventilated precast shear walls with emphasis on sleeve connection efficiency

**DOI:** 10.1038/s41598-025-15816-w

**Published:** 2025-08-26

**Authors:** Hemanth Kumar Anbu, Akash J, Renchana Nair R, Karthikeyan Kothandapani

**Affiliations:** School of Civil Engineering, Vellore Institute of Technology, Chennai, India

**Keywords:** Sleeve connection, Precast structure, Ventilated system, Engineering, Materials science

## Abstract

Precast concrete walls are widely used in modular construction due to their speed of assembly and structural efficiency. However, the presence and size of openings can significantly influence their seismic performance. This study investigates the effect of opening dimensions on the seismic behavior of precast wall specimens subjected to cyclic lateral loading, with a focus on failure mechanisms, hysteretic response, load capacity, and deformability. Two groups of specimens were evaluated: Group A with precast wall (PW-6) 600 mm wide and Group B (PW-4) with 400 mm wide. The experimental results revealed a three-stage failure mechanism, where Group A experienced pronounced diagonal cracking due to reduced confinement, while Group B showed more localized crushing near the opening edges. Hysteresis behavior indicated greater pinching and reduced energy dissipation in Group A, whereas Group B exhibited fuller loops with better energy absorption. Group A’s initial stiffness of 25.6 kN/mm declined to 9.8 kN/mm at failure, while Group B maintained higher stiffness retention and achieved a 12% higher peak lateral load. Ductility ratios were also superior in Group B compared to Group A, highlighting the critical role of confinement and detailing. The objective of this study is to quantify the influence of opening size on seismic response, and the methodology involved reduced-scale cyclic testing of precast wall panels with consistent reinforcement but varying opening widths. The findings provide valuable design insight for enhancing the seismic resilience of precast walls in high-risk zones.

## Introduction

In today’s fast-paced construction environment, the demand for innovative, sustainable, and cost-efficient building solutions has never been greater. Precast Concrete Shear Walls (PCSWs) have emerged as a transformative solution, offering numerous advantages in terms of construction speed, structural performance, and quality assurance. Central to the success of these systems are the connection mechanisms that bind individual precast components into a unified, resilient structure. These connections are engineered to ensure effective force transfer between elements, providing the necessary strength, ductility, and continuity to withstand dynamic loads such as wind and earthquakes. As urbanization accelerates and infrastructure demands intensify, the construction industry increasingly relies on PCSW systems to deliver robust, durable, and adaptable structures.

Precast reinforced concrete (RC) shear walls have become an essential component in modern prefabricated construction due to their efficient fabrication, quality control, and improved construction speed. A wide variety of connection types, such as socket joints^1,2^, headed bar overlaps^3^, grouted tenons^4^, and unjointed vertical bars^5^, have been developed to enhance the seismic behavior of these walls. Despite these advancements, the overall performance of precast systems remains highly dependent on the integrity and ductility of their connections, especially under cyclic loading.

Several studies have evaluated how joint tolerances^6^, frictional interfaces^7^, and axial-tension coupling^8^ affect the deformation capacity and energy dissipation of precast wall systems. Full-scale testing has provided insights into system-level behaviors and deformation mechanisms^9^, while novel connection solutions, such as bundled bars^10^, replaceable energy dissipators^11^, and grout-sleeve joints^12^, have gained traction in addressing performance limitations. The performance and reliability of PCSWs are significantly influenced by the type and configuration of their connection mechanisms. Innovations such as socket connections, Confined Headed Bar Connections (CHBC), and bundled reinforcements have been developed to improve load transfer, energy dissipation, and structural integrity. For instance, socket connections are particularly effective in transmitting shear forces, with failure often localized at the joint root rather than the horizontal interface. This design ensures efficient shear stress transfer and enhances the overall stability of the system. Similarly, CHBC connections provide enhanced confinement, reducing buckling and improving hysteresis behavior under seismic loading, surpassing the performance of traditional cast-in-situ methods. Additionally, factors like interface roughness and socket depth play a critical role in the wall’s load-bearing capacity, with rougher surfaces and deeper sockets contributing to greater peak load resistance. Additionally, studies have investigated special configurations including squat walls^13^, angle-steel connectors^14^, viscoelastic dampers^15^, and hybrid reinforcement strategies^16^.

However, one persistent challenge in precast wall design lies in managing the complex interaction between grouted sleeve connections and openings, especially at critical wall-to-wall junctions. While grouted sleeves offer construction convenience and enable rapid on-site assembly, their performance under lateral cyclic loads is highly sensitive to workmanship quality, material continuity, and confinement^12,17,18^. Several researchers have proposed Ultra-High-Performance Concrete (UHPC)^19^, steel plate bolting^20^, and tooth-groove mechanisms^21^ to address sleeve deficiencies, but issues such as bar misalignment, grouting voids, and joint slip remain difficult to control. Material properties and interface conditions are fundamental to the seismic performance of PCSWs. High-performance materials, such as UHPC, when utilized at boundary elements, significantly enhance the ductility and energy absorption of the walls. Innovations in connection technologies, such as angle steel connectors and spiral-confined lap splices, have also demonstrated improved load-bearing capacity and durability. Reinforced tenons and supplementary V-ties further optimize strength and stiffness, with specific configurations tailored to maximize seismic performance^22,23^.

Moreover, wall openings often required for functional and architectural reasons, can severely disrupt the load path and reduce lateral stiffness and strength^24–26^. They induce localized stress concentrations, leading to premature cracking and reduced ductility in both monolithic and precast configurations^27,28^. Even when combined with advanced assembly methods such as double precast edge members^29^, steel section sleeves^30^, or lightweight concrete^31^, these features pose notable challenges in achieving uniform seismic performance.

Although partial cast-in-place (CIP) regions and modular configurations^32,33^ have been explored, the integration of sleeve joints within critical wall-to-wall interfaces that also include structural openings remains under-investigated. Prior works have typically examined these variables in isolation or with limited emphasis on their coupled effects^33,33–36^. Other studies focus on large-scale walls or alternative connection methods (e.g., bolted-plate^37^, unconnected bars^38^, and horizontal joints^39,40^), but often neglect the complex cracking patterns and failure mechanisms observed when openings intersect joint zones^41,42^.

Despite the extensive research on precast shear wall systems, the coupled influence of sleeve joint behavior and wall openings on seismic performance, particularly at wall-to-wall junctions, remains insufficiently explored. This knowledge gap is critical because such configurations are common in modern modular housing and infrastructure but are prone to brittle failure without adequate confinement and detailing.

This study aims to fill this gap by conducting a comprehensive experimental investigation on the seismic performance of precast concrete shear walls with sleeve connections and integrated openings. By examining different cross-sectional configurations this work evaluates crack propagation, drift capacity, and failure modes across key loading stages (yield, peak, post-peak). Particular attention is given to the interaction between grouted sleeve joints and stress concentrations near openings, highlighting how confinement detailing and reinforcement anchorage affect ductility and energy dissipation.

## Experimental program

### Assembly of specimen

**Fig. 1:**
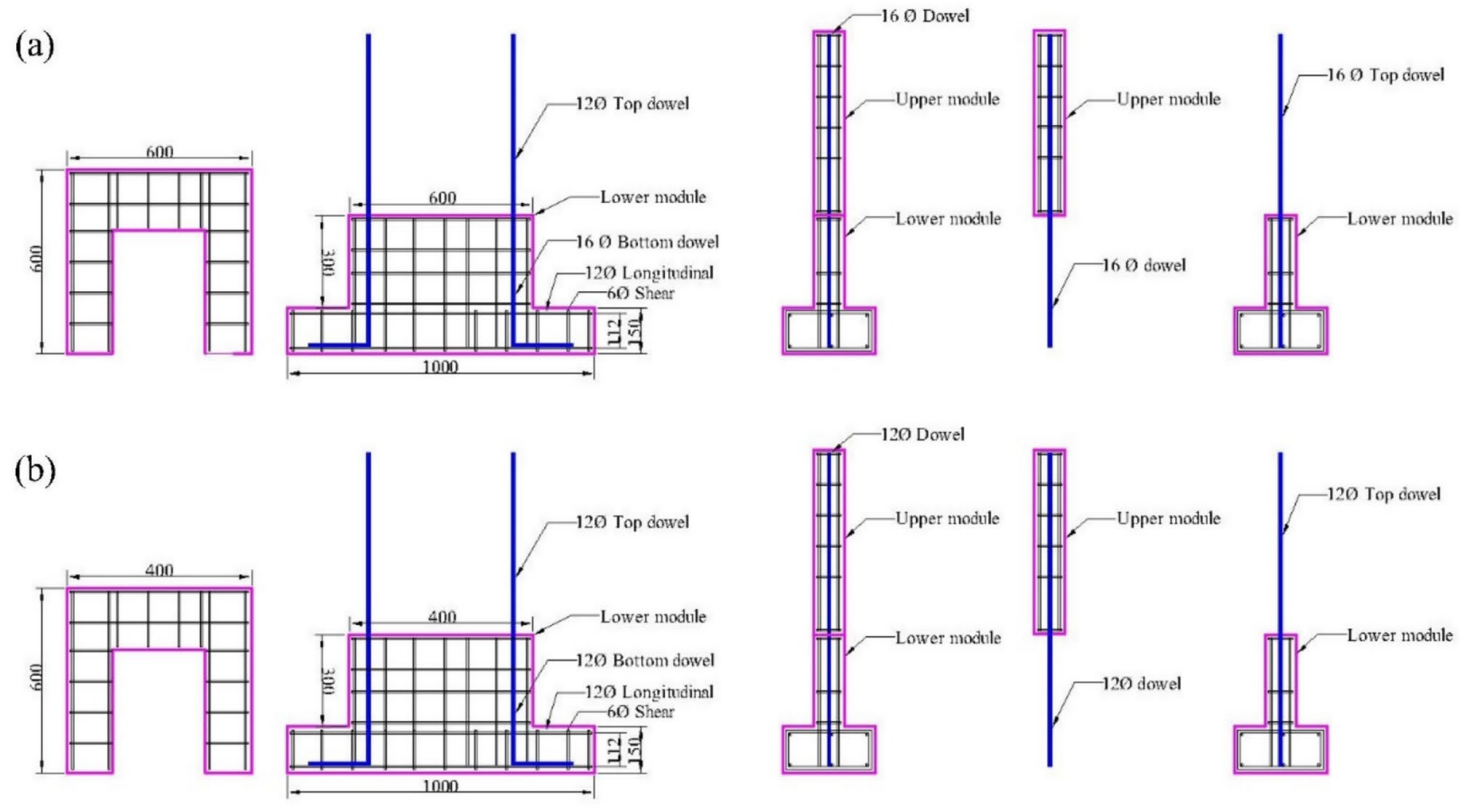
Module positioning: (**a**) PW-600 and (**b**) PW-400.

**Fig. 2:**
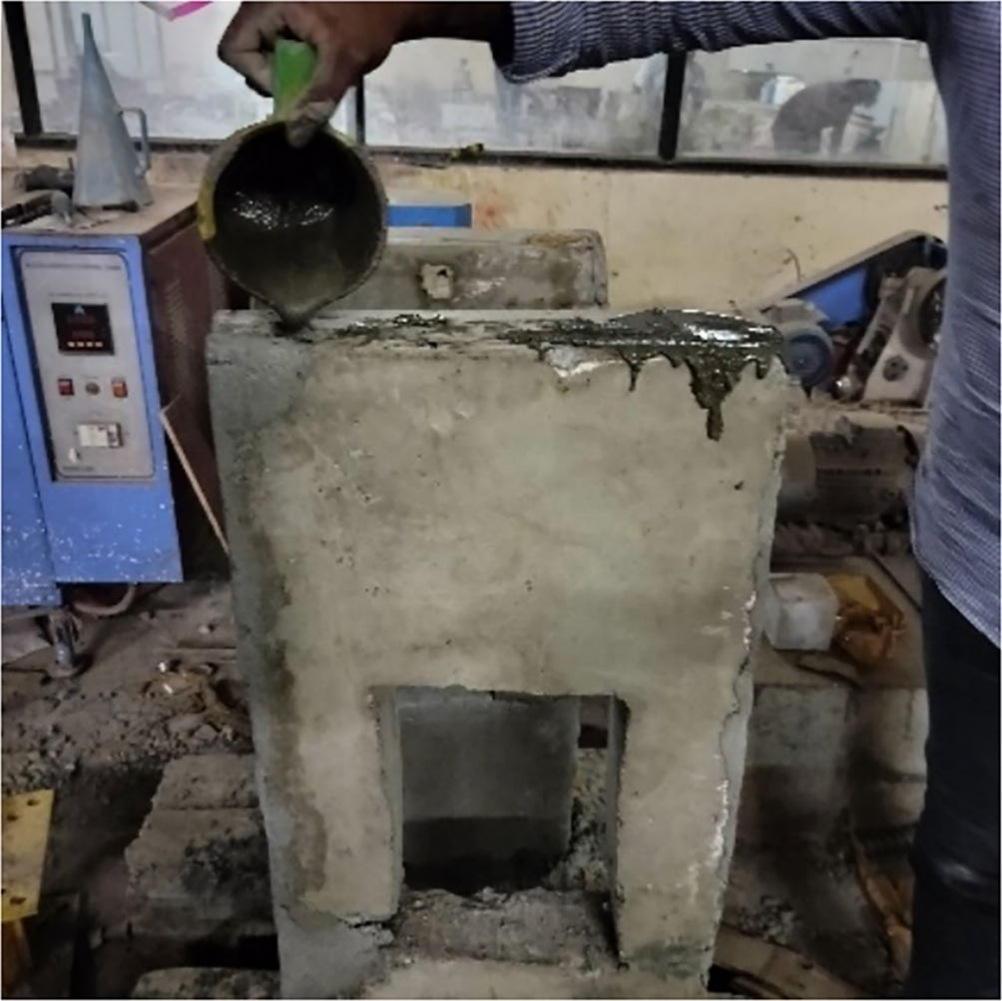
Sleeve grouting.

The integrity and reliability of reinforcing bar connections are paramount in structures subjected to heavy loads and dynamic forces. The precast wall assembly employed a dowel-based connection system, as detailed in the structural drawing presented in Fig. 1. Dowels projecting from the lower module were designed to align and interlock with longitudinal holes in the upper module, ensuring precise engagement. The longitudinal holes in the upper module were integrated with steel sleeves during construction to facilitate accurate positioning and secure seating of the two modules. Following module alignment, the steel sleeve voids were filled with M80 grade grout as shown in Fig. 2, a high-strength material chosen for its superior compressive and bonding properties. This grouting process ensured robust interconnection and continuity across the structural elements, minimizing potential weak points under dynamic loading conditions. The detailed dimensions of the specimens used in the study are provided in Table 1, offering a comprehensive reference for evaluating performance characteristics. This assembly method highlights a systematic approach to achieving structural stability and load transfer efficiency in precast wall systems.

**Table 1:** Specimen dimensions.

Element	Module	Dimension (mm)			Opening (mm)	
Wall		**Length**	**Height**	**Thickness**	**Length**	**Height**
	Upper	600	600	100	200	400
	Lower	600	300	100	-	-
Base		**Length**	**Width**	**Depth**	-	-
		1000	300	150	-	-
Wall		**Length**	**Height**	**Thickness**	**Length**	**Height**
	Upper	400	600	100	200	300
	Lower	400	300	100	-	-
Base		**Length**	**Width**	**Depth**	-	-
		800	300	150	-	-

### Specimen design

The specimens were designed considering the IS 13920: 2016. The slender and intermediate wall was selected from IS 13920: 2016 clause 10.1.4 (h/w > 2 and 1 ≤ h/w ≤ 2 respectively) with a reduced scale of 0.3^39–42^.

*Note: Where “h” and “w” are the height and width of wall respectively.

$$\frac{Height of the wall}{Width of the wall}=\frac{900}{400}=2.25, (Slender)$$

$$\frac{Height of the wall}{Width of the wall}=\frac{900}{600}=1.5, (Intermediate)$$

Two curtains of reinforcements were incorporated into the wall’s cross-section to provide resistance to shear failure. The walls were designed with the factored shear stress demand (Eq. 2) exceeding 0.25√fck (clause 10.1.7), and the ultimate shear was calculated using Eq. 1 (IS 456: 2000, Clause 40.4). The shear strength of walls with openings were checked at critical horizontal planes passing through openings and additional steel reinforcements were provided along all four sides of the openings.

1 $$Vu= \frac{0.87*fy*Asv*d}{Sv}$$

2 $$V= \frac{Vu}{b*d}$$

*Note: Where, fy, Asv, d, Sv, Vu, and V, are yield stress of steel, area of shear reinforcement, depth of wall, provided spacing, ultimate shear strength, Shear stress demand respectively.

### Material properties

The accompanying Fig. 1 shows the dowel reinforcements and associated proportions. For concrete, two grades are specified: M25, commonly used for standard structural applications, and M80, representing high-strength grout typically used for concealing the sleeves as shown in Fig. 2. The elastic modulus of M25 concrete is 25,000 MPa, while the M80 grout has a significantly higher modulus of 44,721 MPa. Both materials exhibit a similar density of 25 kN/m^3^, and a Poisson’s ratio of 0.18, which describes the lateral strain response to compression.

Steel reinforcement of Fe550 grade, has an elastic modulus of 200,000 MPa with a density of 7850 kN/m^3^, the steel has a Poisson’s ratio of 0.30, a typical value for ductile materials, allowing for reliable load redistribution in structural applications. These material properties form the foundation for designing and assessing the performance of structural components under various loading conditions.

### Experimental test setup and loading protocol

**Fig. 3:**
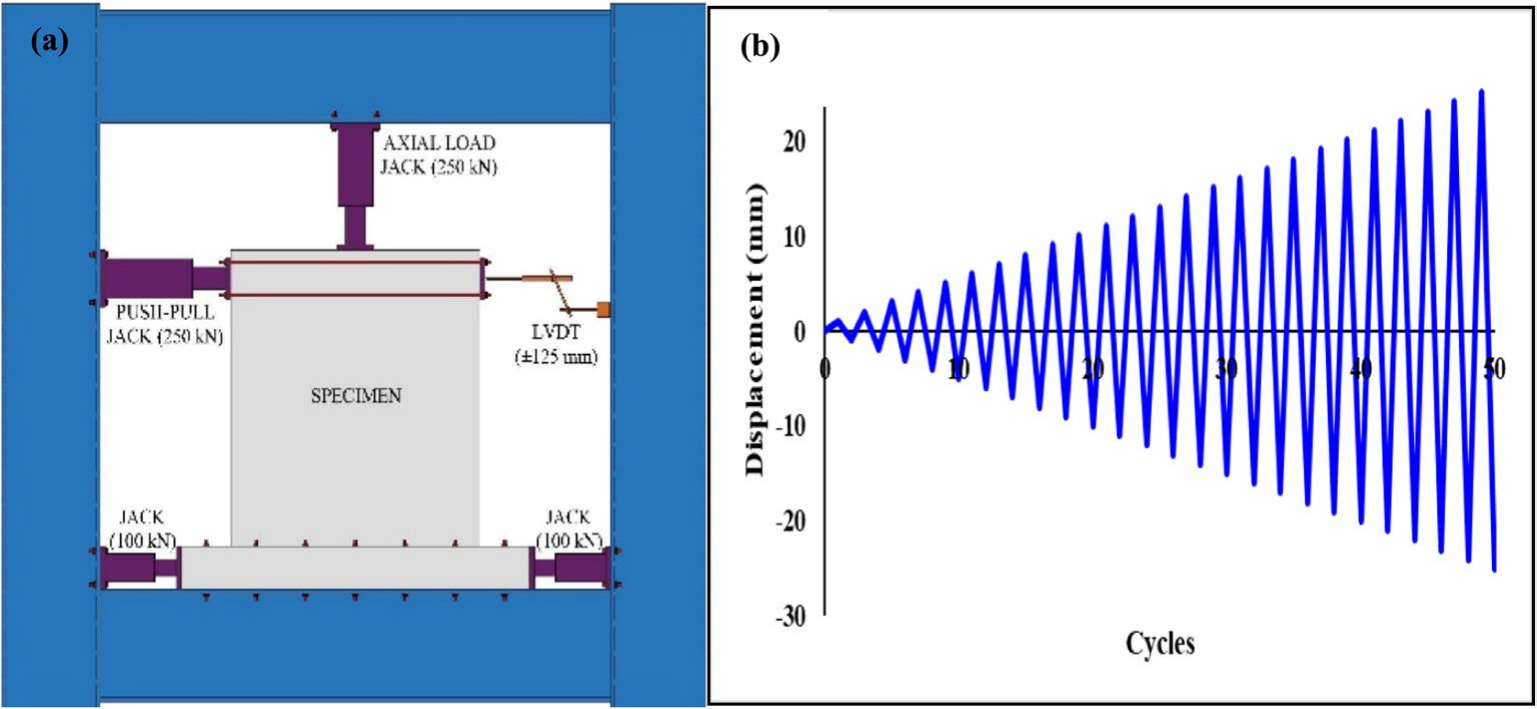
(**a**) Illustrating of test setup and (**b**) Displacement history.

To evaluate the efficacy of precast shear walls and their connections, a reverse cyclic loading test was conducted. A precast shear wall was assembled, with the base securely anchored using jacks and steel flats. Lateral loads, simulating controlled displacements, were applied at the top of the wall using a 25-tonne hydraulic jack as shown in Fig. 3(a). The wall’s response was meticulously monitored using an LVDT to measure displacement at the top of the wall and a load cell to capture the corresponding load. An axial load of 15 kN was applied at the top of the wall in accordance with the provisions of IS 456: 2000. The specimens were subjected to a horizontal force acting perpendicular to its plane. The axial load was considered less than 0.01*fck*Ag as per provision, where fck is the characteristic compressive strength of the concrete, and Ag is the gross cross-sectional area of the wall. This ensured that the applied axial load remained within the specified limits for structural integrity and performance^43–46^.

The specimen was subjected to reverse cyclic loading to simulate the bidirectional forces typically encountered during an earthquake. The loading protocol involved the application of cyclic loads with progressively increasing amplitudes in both the positive and negative directions. The loading amplitude was designed to incrementally increase with each cycle, allowing for a thorough evaluation of the specimen’s performance under various displacement levels. The displacement history for each time interval is detailed in the Fig. 3(b), illustrating the progression of amplitude over the course of the test.

## Experimental observation

### Failure mode

**Fig. 4:**
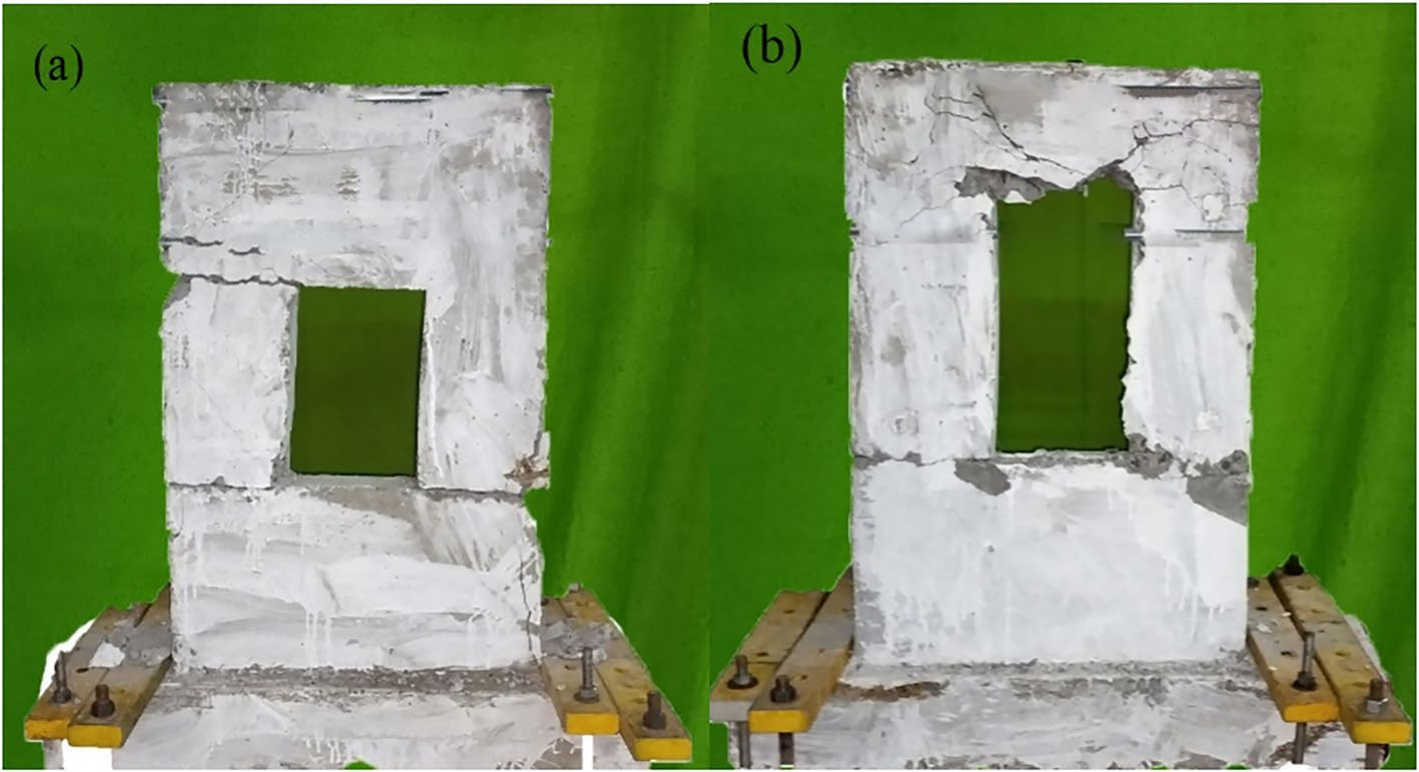
Crack development in: (**a**) PW-6-(200X300) and (**b**) PW-6-(200X400).

**Fig. 5:**
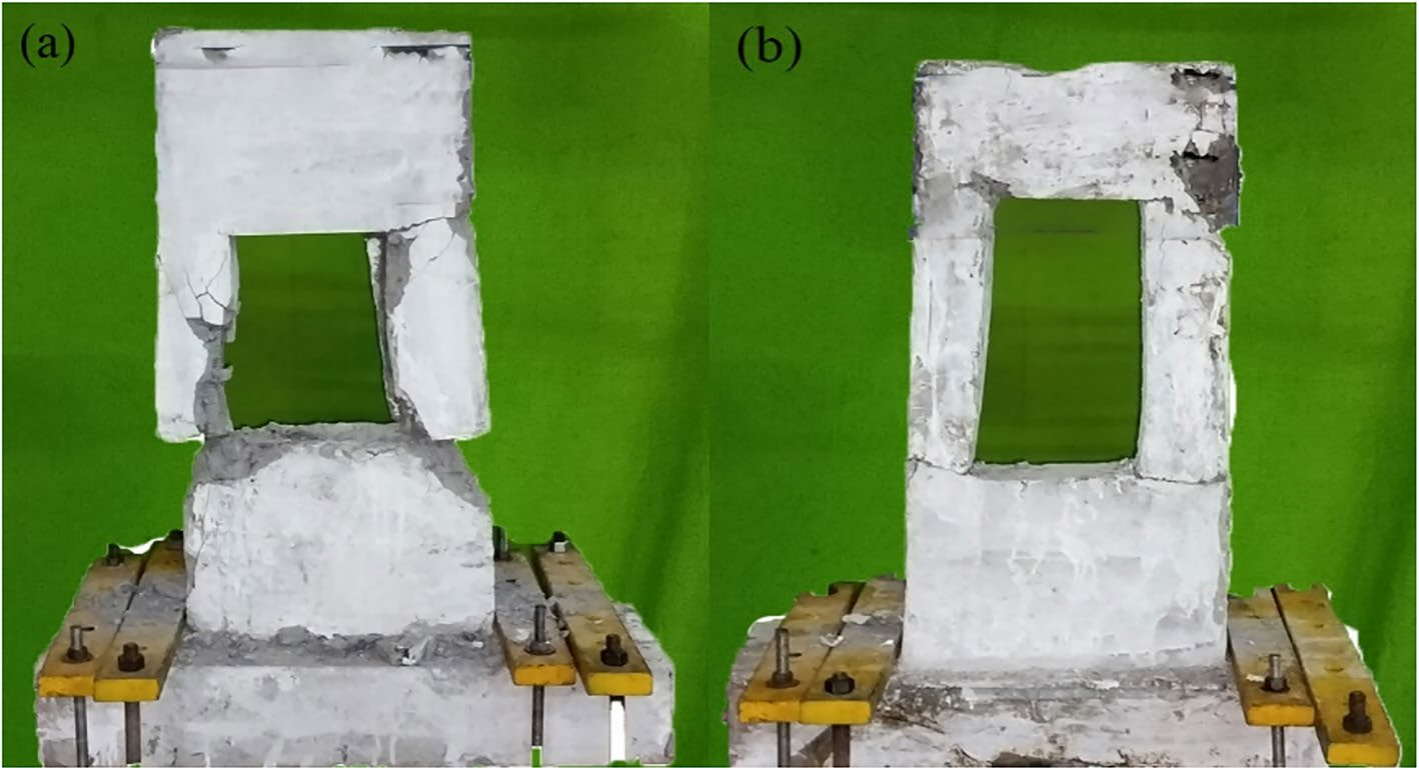
Crack development in: (a) PW-4-(200X300) and (a) PW-4-(200X400).

The failure process of all tested specimens followed a four-stage progression: initial cracking, rebar yielding, peak loading stage, and post-peak behavior. Fig. 4 and Fig. 5 depict the cracking and crushing patterns observed in the concrete of the specimens at the conclusion of the tests. Notable differences were identified among the specimens as the size of the openings varied, as listed in Table 2. These differences are discussed in detail, focusing on Group A (600 mm wide walls) and Group B (400 mm wide walls).

**Table 2:** Drift ratio.

Specimen	δy (%)	δp (%)	δu (%)
PW-6-(200X400)	0.16	1.75	3.39
PW-6-(200X300)	0.28	1.78	2.28
PW-4-(200X400)	0.21	1.51	3.76
PW-4-(200X300)	0.69	1.94	3.01

*Note where 200X400 and 200X300 states the opening sizes in the walls.

#### Group A

##### Rebar yielding

Cracks initiated at the wall-to-wall junction at a drift ratio of 0.16% to 0.28%, exhibiting notable differences between the specimens during this stage. PW-6-(200×400) reached yield at a lower drift ratio (0.16%) compared to PW-6-(200×300) (0.28%). This earlier yielding indicates that the larger specimen responded more quickly to applied lateral forces, due to more efficient force distribution through the increased transverse reinforcement and larger cross-section. Despite earlier yielding, this did not lead to premature failure, rather, it provided controlled energy dissipation from the onset of loading. Conversely, the delayed yielding in PW-6-(200×300) suggests that the stress concentration was higher before reinforcement engagement, leading to a more brittle initial response.

##### Peak stage

Both specimens reached peak strength at approximately the same drift level, 1.75% for PW-6-(200×400) and 1.78% for PW-6-(200×300), indicating that the lateral displacement capacity required to achieve maximum resistance was relatively uniform. However, qualitative differences in crack development during this stage are notable. Fig. 4 illustrates the cracking patterns of both specimens. PW-6-(200×400) exhibited a denser crack network with significant diagonal cracking into the wall panel, a positive indicator of stress redistribution and effective load sharing through dowel connections. PW-6-(200×300), on the other hand, showed horizontal crack extension, implying localized failure mechanisms and limited reinforcement interaction across the lapping zone.

##### Post-peak stage

No new cracks were observed in specimen PW-6-(200×300) post-peak load. At this stage, concrete crushing and spalling occurred, primarily concentrated above the opening in PW-6-(200×400) (Fig. 3). However, the precast panels in PW-6-(200×400) remained relatively intact during loading, indicating effective utilization of the stirrups. The lap length in the vertical connections did not significantly affect the load cycles or the failure modes of PW-6-(200×400) and PW-6-(200×300). The most difference lies in the ultimate drift ratio: PW-6-(200×400) reached 3.39%, significantly outperforming PW-6-(200×300), which failed at 2.28%. This demonstrates the superior ductility and deformation capacity of the former, attributed to increased confinement by stirrups and improved crack control. The additional 1.11% drift capacity translates into greater resilience under seismic loads, highlighting the role of reinforcement detailing in extending the structure’s ability to undergo large deformations before failure.

#### Group B

##### Rebar yielding

PW-4-(200×400) reached the yield stage at a significantly lower drift ratio (0.21%) compared to PW-4-(200×300) (0.69%), indicating a 228% increase in drift before yielding for the latter. This substantial difference highlights a delayed engagement of reinforcement in the narrower specimen, suggesting higher initial stiffness but also increased susceptibility to stress concentration prior to reinforcement activation. In contrast, the earlier yielding of PW-4-(200×400) suggests better load sharing and deformation compatibility due to its larger cross-sectional area and more effective transverse confinement. This early response is advantageous for energy dissipation under seismic demands, as it allows the structure to deform plastically before reaching critical stress levels^47,48^.

##### Peak stage

Both specimens reached peak load at moderate drift ratios: 1.51% for PW-4-(200×400) and 1.94% for PW-4-(200×300). Despite the higher δp for the narrower specimen, its response was governed by more localized damage, especially at the horizontal joint and around the opening. The crack propagation in PW-4-(200×300) concentrated around stress risers, including the wall-to-wall connection zone and the lower corner of the opening, indicating a less uniform stress distribution. On the other hand, the broader and more distributed cracking in PW-4-(200×400) suggests a better integration of reinforcement, resulting in reduced peak stress intensity at critical interfaces.

##### Post-peak stage

At ultimate load, PW-4-(200×400) achieved a higher drift ratio of 3.76%, compared to 3.01% for PW-4-(200×300). This confirms that PW-4-(200×400) had superior deformation capacity and ductility, which can be attributed to the enhanced confinement provided by denser transverse reinforcement and larger section dimensions. The increased ductility not only reflects improved energy dissipation capacity but also signifies greater resilience during post-peak cyclic loading, a crucial requirement for seismic-resistant design. The lower δu in PW-4-(200×300) also aligns with the observed extensive spalling and crushing at the connection zone, indicating premature material degradation and loss of integrity.

### Hysteresis curve

**Fig. 6:**
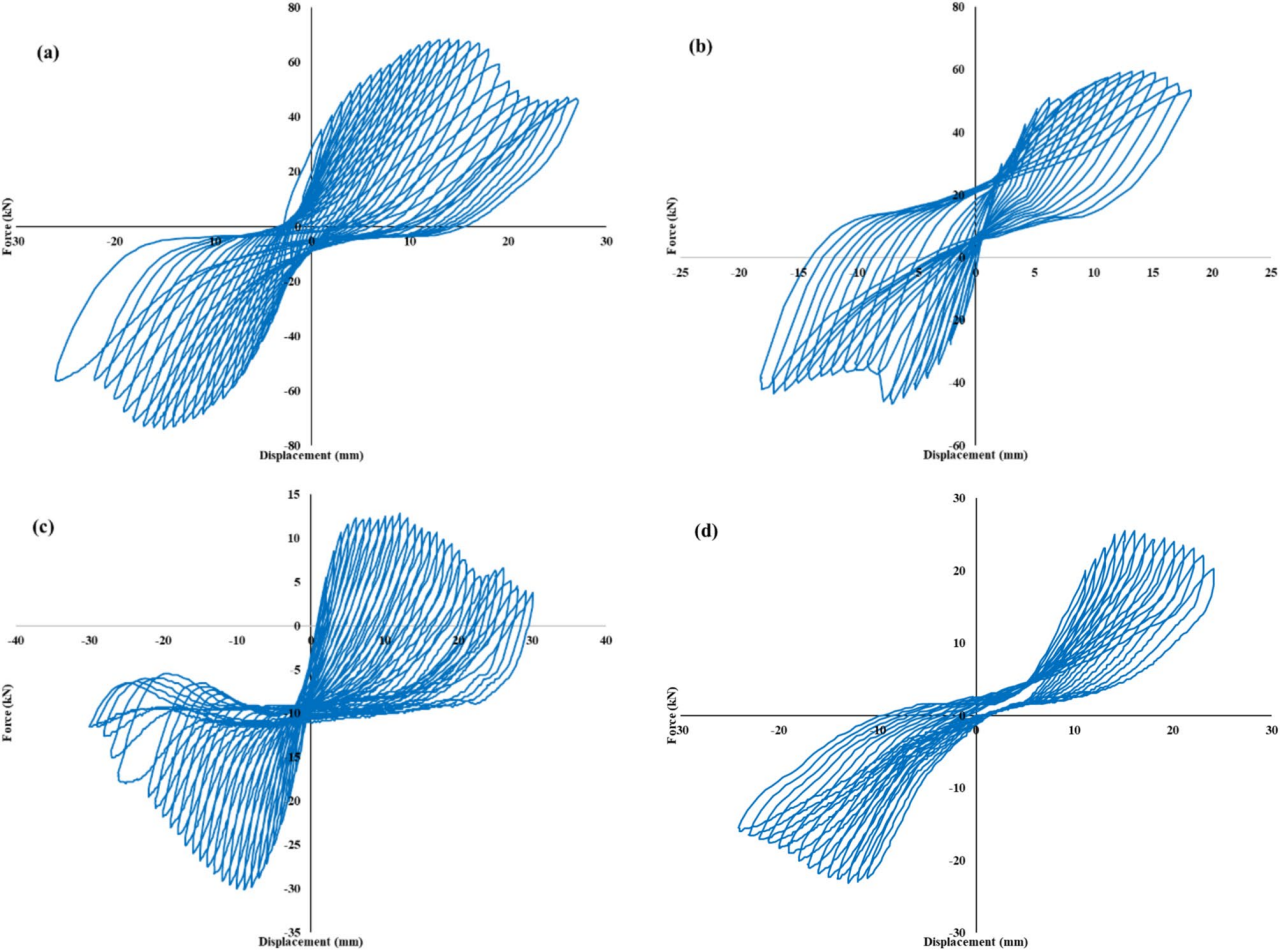
(**a**) PW-6-(200X300), (**b**) PW-6-(200X400), (**c**) PW-4-(200X400) and (**d**) PW-4-(200X300).

The hysteresis behavior of the shear wall specimens shown in Fig. 6 illustrates the cyclic response of hybrid rocking wall systems with different dowel configurations. The test results demonstrate notable variations in energy dissipation and stiffness degradation, which are directly influenced by the number and arrangement of dowels embedded at the base. Specimens PW-6 (Fig. 6(a) and 6(b)), configured with dowels in 200×300 mm and 200×400 mm walls respectively, exhibit relatively broad and stable hysteresis loops. These loops are indicative of effective energy dissipation, minimal pinching, and a more resilient structural response under repeated lateral loading. The stable loop shape and retention of stiffness across cycles highlight the enhanced self-centering capacity and rocking control provided by the increased dowel connectivity.

The PW-400 specimens (Figs. 6(c) and 6(d)), show narrower loops with distinct pinching effects and significant stiffness degradation in later cycles. This behavior reflects reduced energy dissipation capacity and a lower degree of confinement, resulting in compromised load transfer mechanisms at the wall-to-foundation interface. The increased deformation and hysteresis loop contraction observed in these specimens suggest progressive bond deterioration and limited rocking recovery due to insufficient dowel anchorage.

These experimental trends are consistent with the analytical findings of Sadeghi et al. (2023)^49^, who developed a predictive method for evaluating the hysteresis behavior of hybrid rocking walls. Their study emphasized the importance of dowel number and distribution in controlling cyclic stiffness, pinching behavior, and energy dissipation. As supported by both the image data and analytical insights, optimizing the dowel layout significantly improves the seismic resilience of precast hybrid walls by enhancing both energy absorption and re-centering abilities during strong ground motions.

### Skeleton curves

**Fig. 7:**
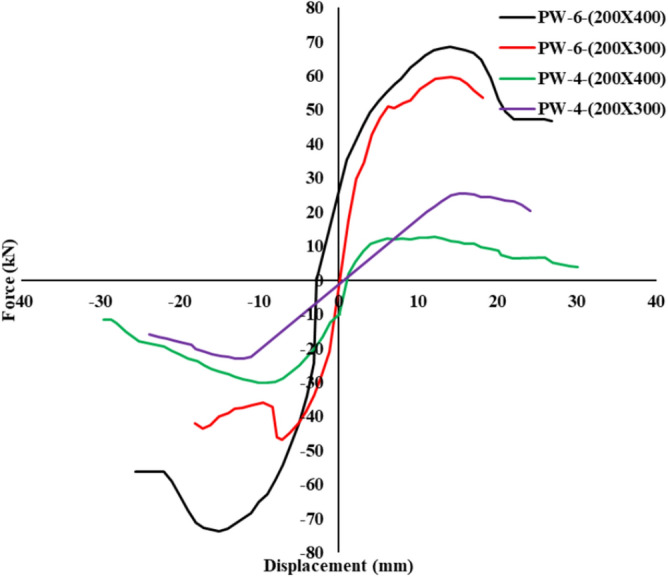
Skeleton curve.

The envelope of the hysteresis curve, referred to as the skeleton curve, is illustrated in Fig. 7 for all specimens. The skeleton curves highlight the critical characteristics of load-displacement behavior under cyclic loading. The key observations are summarized as follows:

#### pre-yielding stage

In the elastic range, the skeleton curves approximated straight lines, indicative of elastic structural behavior. The initial stiffness of PW-6-(200×400) and PW-6-(200×300) was observed to be 5.5% - 48.8% greater than that of PW-4-(200×400) and PW-4-(200×300).

#### Post-peak behavior

The softening trend observed in the skeleton curves beyond the peak load was closely associated with the performance of the horizontal connections:

Group A: The skeleton curves exhibited a steeper softening slope compared to those of Group B. This behavior can be attributed to the influence of the grouting layer, which was extensive in Group A specimens. While the skeleton curves of Group A specimens maintained higher load levels than those of Group B, they experienced faster load degradation after the peak. However, the confinement provided by strengthening measures in the vertical lapping zone delayed concrete crushing, enabling Group A specimens to sustain greater drift values than their Group B counterparts.

Group B: The skeleton curves showed a more abrupt decline in load-carrying capacity after the peak load, primarily due to severe concrete crushing and spalling. This rapid degradation underscores the limited effectiveness of horizontal connections in resisting post-peak forces compared to the more robust configurations in Group A.

The comparative analysis highlights the superior drift capacity of Group A specimens due to delayed crushing and enhanced connection performance, affirming the influence of vertical reinforcement and grouting strategies on structural resilience.

## Evaluation of seismic performance

### Load capacity

The load capacity of the precast wall specimens (PW-6 and PW-4) under cyclic loading was analyzed at the yield, peak, and ultimate stages as listed in Table 3. Percentage differences were calculated to compare the load-carrying performance between specimens of different dimensions and loading cycles, revealing significant insights into the structural behavior.

**Table 3:** Characteristic points of skeleton curves.

Specimen	Cycle	Yield Stage		Peak Stage		Ultimate Stage		Mean Value of μ
		**Load (Fy)**	**Displacement** **(δy)**	**Load (Fp)**	**Displacement** **(δp)**	**Load (Fu)**	**Displacement** **(δu)**	
PW-6-(200X400)	+	34.80	1.30	68.50	14.00	45.30	27.10	12.55
	-	−24.50	−2.90	−73.80	−15.00	−56.30	−25.60	
PW-6-(200X300)	+	29.10	2.20	59.60	14.20	52.50	18.20	8.44
	-	−23.50	−2.10	−46.80	−7.10	−42.10	−18.10	
PW-4-(200X400)	+	1.80	1.70	12.80	12.10	3.50	30.10	16.03
	-	−16.60	−2.00	−30.10	−9.10	−11.50	−29.20	
PW-4-(200X300)	+	4.50	5.50	25.50	15.50	20.00	24.10	5.69
	-	−5.00	−2.90	−23.10	−13.00	−15.80	−23.70	

#### Yield load capacity

The observed variations in yield load capacity (Fy) between the PW-6 and PW-4 specimens can be attributed primarily to changes in sectional geometry and reinforcement detailing. For the PW-6 series, the specimen PW-6-(200x400) demonstrated a higher positive yield capacity of 34.80 kN compared to 29.10 kN for PW-6-(200x300), marking a reduction of approximately 16.4%. Similarly, the negative yield capacity decreased from −24.50 kN to −23.50 kN, a reduction of around 4.1%. This behavior suggests that the reduced section depth from 400 mm to 300 mm significantly lowers the flexural capacity due to a decreased internal lever arm, affecting the moment resistance. The positive yield direction, likely governed by tensile reinforcement behavior, shows more sensitivity to depth reduction, whereas the negative yield response is less affected, possibly due to compression zone behavior and confinement conditions.

The PW-4 specimens show a different trend. The narrower PW-4-(200x300) specimen exhibits a 60.0% increase in positive yield capacity (4.50 kN vs. 1.80 kN) but a drastic 69.9% decrease in negative yield capacity (−5.00 kN vs. −16.60 kN) compared to PW-4-(200x400). This sharp improvement in positive direction performance suggests that reinforcement detailing in the reduced section may have enhanced tensile capacity, such as improved anchorage or bar engagement. However, the severe drop in negative capacity implies potential issues such as reduced confinement, early spalling of cover concrete, or ineffective compression reinforcement.

#### Peak load capacity

The variations in peak load capacity (Fp) among the tested specimens highlight the influence of sectional geometry and reinforcement effectiveness on structural performance under lateral loading. At the peak stage, representing the maximum load a specimen can withstand before significant degradation, PW-6-(200x400) shows higher capacities with Fp values of 68.50 kN in the positive direction and −73.80 kN in the negative. In comparison, the narrower PW-6-(200x300) reaches Fp=59.60 kN and −46.80 kN, indicating a 13.0% reduction in positive peak capacity and a more pronounced 36.6% decrease in negative peak capacity. This discrepancy suggests that the reduction in section depth substantially weakens the specimen’s ability to resist lateral forces, especially in the negative direction. The reduced lever arm in the smaller section limits moment resistance, and potentially poorer confinement or compression zone stability may lead to earlier degradation under reversed cyclic loading.

The PW-4 series further illustrates the complexity of geometry and detailing effects. Interestingly, PW-4-(200x300) shows a 99.2% increase in positive peak capacity (25.50 kN vs. 12.80 kN) compared to PW-4-(200x400), indicating a significant improvement due to more effective tensile reinforcement anchorage or optimized stress flow in the smaller section. Conversely, the negative peak capacity decreases by 23.2% (−23.10 kN vs. −30.10 kN), which may stem from reduced cross-sectional area in the compression zone, leading to earlier crushing or buckling of reinforcement under negative loading. This performance asymmetry reflects how certain configurations may inadvertently favor one loading direction over the other due to detailing practices or confinement conditions.

#### Ultimate load capacity

The ultimate load capacity (Fu), which signifies the residual strength of a structural element following yielding and plastic deformation, reveals critical insights into the influence of geometry and reinforcement detailing on post-peak behavior. For the PW-6 specimens, the wider PW-6-(200x400) exhibits Fu values of 45.30 kN and −56.30 kN, while the narrower PW-6-(200x300) attains 52.50 kN and −42.10 kN, respectively. The 15.9% increase in positive ultimate capacity in the narrower specimen may be attributed to more uniform stress distribution and potentially improved engagement of tensile reinforcement. However, the 25.2% reduction in the negative ultimate capacity suggests that compression-related mechanisms, such as concrete crushing or bar buckling, are more sensitive to reduced cross-sectional dimensions and confinement, leading to earlier degradation in strength.

In the PW-4 series, the disparities are even more pronounced. PW-4-(200x400) achieves Fu=3.50 kN and −11.50 kN, whereas PW-4-(200x300) demonstrates significantly improved positive performance with Fu=20.00 kN, a 471.4% increase. This leap in capacity can likely be attributed to better reinforcement utilization and minimized eccentricity or cracking in the narrower section under tension. Nonetheless, the negative capacity drops by 37.4%, indicating that under compressive action, the smaller section is less capable of sustaining plastic deformation, likely due to insufficient confinement, reduced bearing area, and loss of stability in the compression zone.

The overarching trend showed that PW-6 specimens, with larger cross-sections and potentially better detailing, exhibit higher yield, peak, and ultimate capacities than the PW-4 series, reflecting greater load-carrying ability and energy dissipation. Within each series, wider specimens (200x400 mm) generally provide better performance under negative (compressive) loading due to larger concrete volume, improved stiffness, and delayed onset of buckling or crushing. Conversely, narrower specimens (200x300 mm) sometimes outperform in positive (tensile) ultimate capacity, possibly because they experience more uniform strain distribution, fewer crack propagations, and better load path continuity. These findings underscore the complex interplay between geometry, reinforcement strategy, and loading direction in defining structural resilience and post-yield behavior.

These variations can be attributed to several factors, such as differences in reinforcement distribution, concrete damage accumulation, and asymmetrical confinement effects in the wall joints and opening regions. Uneven detailing at the wall-to-wall joints and the possible pre-existing imperfections during specimen fabrication may also lead to directional sensitivity. The presence of an opening, for instance, may localize stresses differently during positive and negative loading cycles, promoting early cracking or crushing depending on the direction. For design, this highlights the critical need to account for such directional imbalances. It suggests that assuming symmetric cyclic behavior in structural models may lead to underestimation of failure risks in one direction. Hence, future designs of demountable or precast systems, especially those involving wall-to-wall joints, must incorporate detailing that ensures balanced performance under bidirectional loading, improving both safety and resilience in seismic applications.

### Displacement ductility and deformability

#### Displacement ductility and deformability for PW-6 specimens

For PW-6-(200x400), the positive cycle shows a yield displacement (δy​) of 1.30 mm and an ultimate displacement (δu​) of 27.10 mm. This results in a displacement ductility factor (μ) of 12.55, which is relatively high, indicating that the specimen can sustain significant deformation after yielding. The large difference between δy​ and δu suggests excellent deformability, contributing to high energy dissipation and providing resilience under seismic loading. In the negative cycle, the specimen exhibits a yield displacement of −2.90 mm and an ultimate displacement of −25.60 mm, with a similar high ductility factor of 12.55, reinforcing its ability to maintain structural integrity through substantial cyclic deformation.

PW-6-(200x300), on the other hand, shows slightly reduced ductility compared to the 200x400 configuration. The positive cycle has a yield displacement (δy​) of 2.20 mm and an ultimate displacement (δu​) of 18.20 mm, resulting in a ductility factor of 8.44. This represents a 33% reduction in displacement ductility compared to the larger specimen. While still exhibiting significant deformability, this specimen is more prone to stiffness degradation and earlier failure compared to the larger configuration. The negative cycle shows a similar trend with a yield displacement of −2.10 mm and an ultimate displacement of −18.10 mm, resulting in a slightly lower ductility factor of 8.44, which is about 33% less than the larger specimen’s ductility factor.

The PW-6 series, representing hybrid-coupled walls with enhanced dowel connectivity, demonstrated considerable deformation capacity. PW-6-(200×400) exhibited a yield displacement (δy) of 1.30 mm and an ultimate displacement (δu) of 27.10 mm in the positive cycle, resulting in a displacement ductility factor (μ) of 12.55. This high ductility is indicative of excellent post-yield deformability and energy dissipation, aligning with the findings of Jafari et al. (2024)^49^, who noted that increased confinement and dowel engagement delay damage progression and enable structures to withstand extensive cyclic loading. In the negative cycle, a similar ductility factor (μ = 12.55) was observed, reinforcing the symmetry and robustness of the wall’s behavior.

Conversely, PW-6-(200×300) exhibited a more modest ductility response, with a yield displacement of 2.20 mm and ultimate displacement of 18.20 mm in the positive cycle, producing a ductility factor of 8.44, a 33% reduction compared to the 200×400 variant. This reduced capacity suggests an earlier onset of stiffness degradation and damage accumulation, particularly near critical regions such as the base joints. The negative cycle mirrored this trend, reflecting the influence of reduced wall width and connection area on the hysteretic response.

#### Displacement ductility and deformability for PW-4 specimens

The PW-4 series, showed even more distinct disparities in ductility. PW-4-(200×400) demonstrated outstanding deformability, with a yield displacement of 1.70 mm and an ultimate displacement of 30.10 mm in the positive cycle, resulting in a very high ductility factor of 16.03. This performance is consistent with the cyclic behavior observed in hybrid-coupled systems studied by Jafari et al. (2024)^50^, where ductile responses were maintained through well-detailed reinforcement and delayed damage propagation. The same ductility factor was observed in the negative direction, highlighting balanced energy dissipation and structural resilience.

PW-4-(200×300) showed a significant drop in ductility, with a factor of 5.69. The yield displacement of 5.50 mm and ultimate displacement of 24.10 mm in the positive cycle reflects a 64% reduction compared to the 200×400 configuration. This steep decline suggests that premature concrete crushing and localized failure dominated its post-yield response, corroborating damage patterns described by Jafari et al., where shear-dominated failure modes in less confined systems led to early strength degradation and reduced hysteretic energy absorption.

#### Comparative analysis of displacement ductility and deformability

Across all configurations, PW-4-(200×400) emerged as the most ductile and damage-tolerant, followed closely by PW-6-(200×400), both displaying high mean ductility factors (μ = 16.03 and 12.55, respectively) and robust hysteresis loops. These findings suggest that increased wall width and dowel quantity effectively delay crack propagation and crushing, preserving load-carrying capacity under severe cyclic loading. In contrast, the 200×300 specimens (both PW-4 and PW-6) exhibited reduced ductility (μ = 5.69 and 8.44), earlier stiffness degradation, and more rapid damage evolution—particularly in PW-4-(200×300), where localized crushing severely impaired cyclic performance.

Stiffness degradation.

**Fig. 8:**
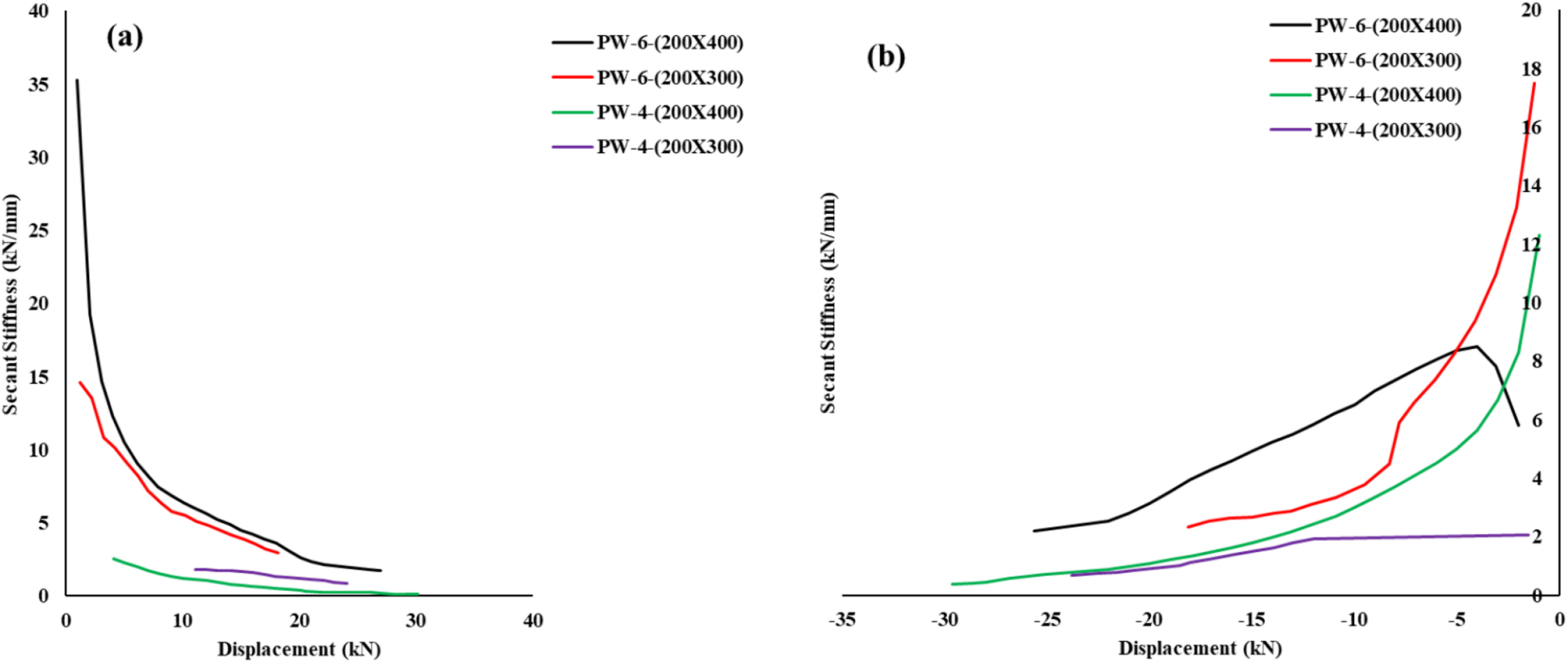
Stiffness degradation: (**a**) Positive cycle and (**b**) Negative cycle.

Fig. 8(a) and 8(b) illustrates the positive and negative stiffness degradation curve respectively of each specimen. The secant stiffness at the peak, Kj, was calculated by Eq. (1).

1 $$Kj= \frac{\sum_{i=n}^{n}{P}_{j}^{i}}{\sum_{i=1}^{n}{\Delta }_{j}^{i}}$$

where ${P}_{j}^{i}$ and ${\Delta }_{j}^{i}$ are the peak load and corresponding lateral displacement at the top at the ith cycle of the jth loading sequence, respectively, and n is the number of cycles. Generally, the lower the stiffness is, the more damage the wall accumulates. Therefore, the degradation of K j with the increase of drift could reflect the damage development of walls under the cyclic load. According to the test results, the characteristics of degradation can be drawn as follows.

Stiffness degradation is a critical parameter in assessing the structural integrity of precast wall specimens under cyclic loading. The progressive reduction in stiffness from the yield to ultimate stages reflects the extent of damage accumulation and the energy dissipation capacity of the structural element. The analysis of the provided data highlights distinct stiffness degradation behaviors in PW-6 and PW-4 specimens, influenced by their dimensions and load conditions.

For PW-6-(200x400), the initial stiffness at the yield stage (ky​) is calculated as 26.77 kN/mm in the positive cycle. This value drops significantly to 4.89 kN/mm at the peak stage, indicating an 81.7% reduction due to the onset of yielding and cracking. By the ultimate stage, stiffness further declines to 1.67 kN/mm, reflecting an additional 65.8% reduction from the peak. In the negative cycle, a similar trend is observed, where ky=8.45 kN/mm, ky​=8.45kN/mm, kp=4.92 kN/mm, kp​=4.92kN/mm (41.7% reduction), and ku=2.20 kN/mmk_u = 2.20, ku​=2.20kN/mm (55.3% reduction from the peak). For PW-6-(200x300), the initial stiffness is 13.23 kN/mm, which reduces to 4.20 kN/mm at the peak stage (68.3% reduction) and further to 2.88 kN/mm at the ultimate stage (31.4% reduction). The negative cycle follows a comparable pattern with ky=11.19 kN/mm, kp=6.59 kN/mm (41.1% reduction), and ku=2.33 kN/mm (64.6% reduction from the peak). These results for PW-6 specimens indicate a rapid loss of stiffness, highlighting the brittle failure mechanisms inherent to these walls.

For PW-4-(200x400), the initial stiffness (ky​) is 1.06 kN/mm in the positive cycle and remains constant at the peak stage (kp=1.06 kN/mm), showing no significant stiffness degradation. However, at the ultimate stage, the stiffness sharply decreases to 0.12 kN/mm, reflecting an 88.7% reduction from the peak. In the negative cycle, ky=8.30 kN/mm, decreasing to kp=3.31 kN/mm (60.1% reduction) and further to ku=0.39 kN/mm (88.2% reduction from the peak). For PW-4-(200x300), the initial stiffness is ky=0.82 kN/mm, which increases to kp=1.65 kN/mm, showing a 100.8% increase due to elastic behavior between yield and peak stages. However, the stiffness decreases to 0.83 kN/mm at the ultimate stage (49.7% reduction from the peak). In the negative cycle, the stiffness transitions from ky=1.72 kN/mm to kp=1.78 kN/mm (3.5% increase) before reducing to ku=0.67 kN/mm (62.4% reduction from the peak). These findings suggest that PW-4 specimens exhibit more gradual stiffness degradation, particularly in PW-4-(200x300), which demonstrates better ductility and energy dissipation characteristics.

The comparison between PW-6 and PW-4 specimens highlights distinct stiffness behaviors under cyclic loading. PW-6 specimens exhibit higher initial stiffness due to their larger cross-sections, offering better initial resistance to loads. However, they also experience more rapid stiffness degradation, underscoring brittle failure characteristics. In contrast, PW-4 specimens, despite having lower initial stiffness, retain their stiffness more effectively, showcasing improved post-yield performance. Notably, the elastic behavior observed in PW-4-(200x300) between the yield and peak stages indicates an optimal reinforcement-to-concrete interaction, making it more suitable for applications requiring enhanced deformation capacity.

The analysis underscores a trade-off between initial stiffness and resistance to stiffness degradation. PW-6 specimens are suitable for applications requiring high initial stiffness but may require reinforcement detailing improvements to mitigate rapid stiffness loss. On the other hand, PW-4 specimens are better suited for seismic applications where ductility and energy dissipation are critical. The findings emphasize the importance of optimizing reinforcement configurations and incorporating advanced materials to control stiffness degradation, thereby enhancing structural resilience and extending service life.

### Energy dissipation capacity

**Fig. 9:**
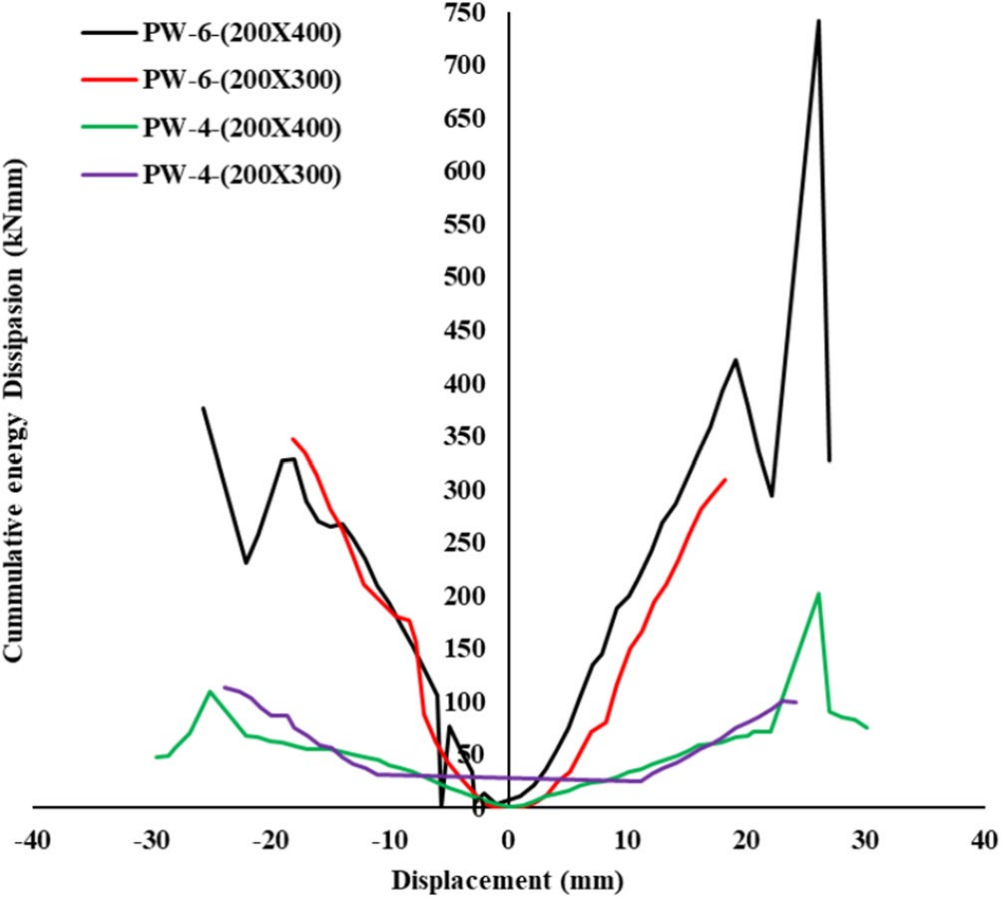
Cumulative energy dissipation.

The energy dissipation performance of precast wall specimens is quantitatively depicted in Fig. 9, with results indicating significant variations across different geometries and reinforcement configurations. As highlighted in^51^, optimal placement and configuration of energy-dissipating components critically influence the hysteretic behavior and deformation capacity of hybrid self-centering walls. In line with these findings, the results from the current study show distinct behavioral patterns among the PW-6 and PW-4 series.

For the PW-6-(200×400) specimen, the hysteresis response under positive cyclic loading yields a peak load (Fp) of 68.50 kN at a displacement (δp) of 14.00 mm, and an ultimate load (Fu) of 45.30 kN at δu = 27.10 mm. The corresponding displacement from yield to ultimate reflects a substantial deformation range. Under negative loading, the specimen reaches a peak load of −73.80 kN and an ultimate load of −56.30 kN at δu = −25.60 mm. The mean ductility factor (μ) of 12.55 demonstrates efficient energy dissipation, consistent with the concept of integrated energy-dissipating mechanisms as outlined by Sadeghi et al. (2024).

The PW-6-(200×300) shows a positive peak load of 59.60 kN, approximately 13% lower than the 200×400 configuration, while achieving a slightly greater δp of 14.20 mm. Despite this reduction in peak strength, its ultimate load rises to 52.50 kN (16% higher), indicating a delayed strength degradation. However, the negative cycle exhibits a significant drop, with peak and ultimate loads falling by 36.6% and 25%, respectively. The mean ductility factor reduces to 8.44, around 33% lower than its 200×400 counterpart, suggesting diminished post-yield deformation and energy absorption capacity.

The PW-4 series, the PW-4-(200×400) shows a notably lower positive peak load of 12.80 kN (81% lower than PW-6-(200×400)) at δp = 12.10 mm. Nonetheless, its ultimate load of 3.50 kN at δu = 30.10 mm signals a large deformation capability. A ductility factor of 16.03, which is 28% higher than the PW-6-(200×400), reflects superior energy dissipation despite its reduced strength, aligning with findings by Sadeghi et al. that deformation-centric mechanisms contribute to energy absorption in hybrid rocking systems. Under negative loading, the peak and ultimate loads drop to −30.10 kN and −11.50 kN, respectively, but still demonstrate capacity for extended deformation.

The PW-4-(200×300) specimen achieves a positive peak load of 25.50 kN (62.8% lower than PW-6-(200×400)) at δp = 15.50 mm, with an ultimate load of 20.00 kN at δu = 24.10 mm, indicating more limited energy dissipation. The negative cycle presents further reductions, with peak and ultimate loads of −23.10 kN and −15.80 kN, representing 68.7% and 71.9% reductions, respectively, compared to PW-6-(200×400). The mean ductility factor of 5.69, 55% lower than the reference specimen, reinforces its lower seismic performance.

## Conclusion

The following conclusion synthesize key observation on the structural behavior of precast wall specimens under cyclic lateral loading. Emphasis is placed on cracking patterns, reinforcement effectiveness, and confinement detailing, all of which critically influence ductility, energy dissipation, and post-peak performance. Comparative analysis of specimen groups highlights the role of cross-sectional dimensions and reinforcement density in resisting seismic demands.

1. Cracks initiated at the wall-to-wall junction due to stress concentration, a common occurrence at geometric discontinuities and interfaces between precast elements. For PW-6-(200 × 400), the larger transverse reinforcement reduced stress localization, delaying crack initiation compared to PW-6-(200 × 300). The restraint provided by stirrups mitigates tension-induced cracking, emphasizing the role of reinforcement in controlling early crack propagation.
2. The longitudinal and dowel reinforcements yielded under cyclic tension at the extreme edges where moment demand is highest. The observed horizontal cracks in PW-6-(200 × 300) and fewer cracks in the lapping zone of PW-6-(200 × 400) are due to the confinement effects of transverse stirrups. The enhanced reinforcement density in PW-6-(200 × 400) increased lateral resistance, reducing the extent of cracking.
3. At peak load, crack density and propagation are governed by the balance between tensile stresses and the capacity of the reinforcing system. For PW-6-(200 × 400), the increased crack density reflects a redistribution of stresses due to effective dowel action, which minimizes stress accumulation at the seams. In contrast, horizontal seams in PW-6-(200 × 300) exhibited reduced integrity, leading to broader crack formation.
4. Concrete spalling and crushing above the openings in PW-6-(200 × 400) occur as compressive stresses exceed the concrete’s ultimate strength. The integrity of precast panels in PW-6-(200 × 400) is preserved by stirrups that confine concrete, delaying crushing. In both specimens, residual strain below yield strain indicates the limited role of vertical connection lap lengths in influencing post-peak performance.
5. Initial cracking occurred within the drift range of 0.16–0.28%, directly correlating with the yield drift values. PW-6-(200 × 400), with earlier yield, showed vertical splitting at corners, reflecting a more distributed and ductile stress response, while PW-6-(200 × 300) showed horizontal cracking, indicative of shear concentration and weaker confinement.
6. During the peak stage, although both specimens had similar drift values, PW-6-(200 × 400) developed more distributed cracks, including diagonals, without vertical seam propagation, confirming stronger dowel-bar anchorage and shear continuity.
7. The pronounced pinching and rapid degradation in PW-4-(200 × 300) are due to localized failure at the wall-to-wall connections and the inability of dowels and transverse reinforcement to confine spalling effectively. PW-4-(200 × 400), with its larger transverse reinforcement, maintained a plumper hysteresis loop, highlighting its superior energy dissipation capacity and resistance to progressive failure.
8. In the elastic range, the straight-line approximation indicates that load–displacement behavior is dominated by the stiffness of the reinforcing and concrete systems. The higher stiffness of PW-6 specimens compared to PW-4 specimens is attributed to the larger cross-section and enhanced confinement provided by transverse reinforcements.
9. During initial cracking, the large disparity in δy values (0.21% vs. 0.69%) clearly reflects the difference in stiffness and confinement between the two specimens. The earlier cracking in PW-4-(200 × 400) did not indicate weakness but rather early engagement of reinforcement leading to controlled cracking.
10. At the yielding stage, PW-4-(200 × 400) showed more diagonal cracks originating from wall corners, indicating effective force redistribution through transverse and dowel reinforcements. In contrast, the horizontal cracking and delayed yielding in PW-4-(200 × 300) suggest stress accumulation and localized failure mechanisms.
11. During the peak and post-peak stages, although both specimens developed extensive cracking, the crack distribution in PW-4-(200 × 400) remained more uniform, with better retention of structural integrity. PW-4-(200 × 300) displayed clear signs of progressive failure, including stirrup yielding and crushing, ultimately resulting in a more brittle collapse pattern.
12. The steeper softening slopes of Group A specimens post-peak indicate the extensive grouting layer’s influence, which absorbs and redistributes stresses effectively. In contrast, Group B’s abrupt degradation reflects inadequate horizontal connection capacity to resist cyclic forces, highlighting the structural deficiencies of reduced dowel anchorage and transverse confinement.
13. The higher yield capacity of PW-6-(200 × 400) compared to PW-6-(200 × 300) is due to the increased reinforcement cross-section and better confinement in the former. For PW-4 specimens, the narrower PW-4-(200 × 300) achieves higher positive yield capacity due to improved stress distribution over a smaller area, though negative capacity is reduced due to localized stress concentrations.
14. At peak load, PW-6-(200 × 400) exhibited superior performance due to the optimized reinforcement detailing, which delayed crack propagation and increased load resistance. The reduction in negative peak capacity for narrower specimens (PW-6-(200 × 300) and PW-4-(200 × 300)) is caused by stress localization at the vertical seams, reducing their ability to resist lateral forces.
15. The residual strength at ultimate load is influenced by the energy dissipation and damage accumulation mechanisms. PW-6-(200 × 400) demonstrated better positive ultimate capacity due to the increased confinement, which mitigated post-peak cracking and spalling. The reductions in negative ultimate capacities for narrower specimens reflect their limited ability to redistribute loads post-failure.
16. Asymmetric stiffness and strength degradation under cyclic loads can lead to residual displacements, impacting re-centering capability of the wall system after an earthquake.
17. Designers must consider these behaviors in the nonlinear dynamic analysis of structures, especially in high seismic zones.
18. Detailing improvements such as symmetrical anchorage conditions, enhanced confinement, and balanced reinforcement layouts may mitigate such asymmetries.
19. In precast systems, joint detailing and tolerance control are vital to reduce construction-induced asymmetry.

## Supplementary Information

Supplementary Information.

## Data Availability

The data that support the findings of this study are available on request from the corresponding author.
